# Reliability of Parental Recall of Birth Weight, Birth Length and Gestational Age in the Multicenter Cohort Study IDEFICS

**DOI:** 10.1007/s10995-024-04009-y

**Published:** 2024-10-19

**Authors:** Annika Swenne, Toomas Veidebaum, Michael Tornaritis, Marika Dello Russo, Luis A. Moreno, Dénes Molnár, Staffan Mårild, Stefaan De Henauw, Iris Pigeot, Hermann Pohlabeln

**Affiliations:** 1 Leibniz Institute for Prevention Research and Epidemiology–BIPS, Achterstraße 30, 28359 Bremen, Germany; 2 Department of Chronic Diseases, National Institute for Health Development, Hiiu 42, 11619 Tallinn, Estonia; 3 Research and Education Institute of Child Health, 138, Limassol Ave, #205, 2015 Strovolos, Cyprus; 4 Institute of Food Sciences, National Research Council, Via Roma, 52 A/C, 83100 Avellino, Italy; 5 GENUD (Growth, Exercise, Nutrition and Development) Research Group, University of Zaragoza, Pedro Cerbuna, 12, 50009 Saragossa, Spain; 6 Centro de Investigación Biomédica en Red de Fisiopatología de La Obesidad y Nutrición (CIBERObn), Instituto de Salud Carlos III, Monforte de Lemos 3-5. Pabellón 11. Planta 0, 28029 Madrid, Spain; 7 Instituto Agroalimentario de Aragón (IA2), Calle Miguel Servet, 177, 50013 Saragossa, Spain; 8 Instituto de Investigación Sanitaria Aragón (IIS Aragón), Avda. San Juan Bosco, 13, 50009 Saragossa, Spain; 9 Department of Pediatrics, Medical School, University of Pécs, Szigeti Str 12, 7624 Pecs, Hungary; 10 Department of Pediatrics, Institute of Clinical Sciences, Sahlgrenska Academy at Gothenburg University, Box 100, 40530 Gothenburg, Sweden; 11 Department of Public Health and Primary Care, Faculty of Medicine and Health Sciences, Ghent University, Corneel Heymanslaan 10, 9000 Ghent, Belgium; 12 Faculty of Mathematics and Computer Science, University of Bremen, Post Office Box 330440, 28334 Bremen, Germany

**Keywords:** Birth length, Birth weight, Gestational age, Parental recall, Reliability

## Abstract

**Objective:**

To investigate the reliability of parental recall of birth weight, birth length and gestational age several years after birth.

**Methods:**

Parentally recalled birth parameters were obtained from the European multicentric cohort study IDEFICS (Identification and prevention of dietary- and lifestyle-induced health effects in children and infants) and compared to the corresponding data externally recorded in the child’s medical check-up booklet. The agreement between the two sources was examined using Bland–Altman plots, intraclass correlation coefficients and Cohen’s kappa for clinically relevant categories. Additionally, logistic regression models were used to identify factors related to parental recall accuracy.

**Results:**

A total of 4930 children aged 2 to 11 years were included. Accuracy of birth weight within 100 g was 88%, 94% of the recalled birth length was within 2 cm, and 99% of the parents could recall with 2 weeks accuracy how many weeks their child was delivered preterm. Discrepancies of more than two weeks or more than 100 g were more likely in parents of low or medium socioeconomic status. Non-biological parents were 3.4 times more likely to have a discrepancy of more than 100 g compared to biological mothers (95% CI 1.7–6.7). Moreover, parents were less likely to accurately recall their child’s birth parameters with increasing number of children within a family.

**Conclusions for Practice.:**

In general, parents’ information matched well with the medical check-up booklet. However, accuracy varied among different groups which should be taken into consideration when using birth data recalled by parents in studies of child health.

## Significance

What Is Already Known?

What This Study Adds?

Parentally recalled birth data have been shown to be generally reliable but there is evidence that the accuracy depends on various sociodemographic factors.

Most studies analyzing the reliability of parentally recalled birth data include only limited age ranges of the children and are relatively small. This study comprises a large sample, covers a wide age range of the children and identifies several factors (e.g. interviewed parent, socioeconomic status, number of siblings) that may influence the accuracy of reported information.

## Introduction

In recent years, there has been increasing evidence that the risk for various non-communicable diseases such as cardiovascular diseases, obesity, or ADHD is not only determined by current exposure but can also be attributed to prenatal and early childhood factors (Baird et al., 2017; O'Donnell & Meaney, 2017). In this context, fetal growth is of particular importance as a marker of exposure during pregnancy and as a predictor of future child growth. Fetal growth is often characterized by pregnancy outcomes such as birth weight, gestational age and length at birth. However, records of these birth parameters are not always available, for example if a child was born several years ago. Therefore, the birth parameters are often collected by parental questionnaires. It has been shown, that parentally recalled birth data are generally reliable and of good quality (Bat-Erdene et al., 2013; Shenkin et al., 2017; Skulstad et al., 2017). However, there is evidence that the accuracy depends on various sociodemographic factors. For example, one study in healthy adolescents demonstrated that mothers were more accurate in reporting birth weight than fathers or other legal guardians (Kassem et al., 2018). Another study found that higher socioeconomic status (SES) was associated with a higher accuracy of the maternally-reported infant birth weight information (Tate et al., 2005).

The IDEFICS (Identification and prevention of dietary- and lifestyle-induced health effects in children and infants) cohort provided the opportunity to further investigate factors that might influence accuracy and to gain new insights into the reliability of parental recall of birth parameters based on a large study population. For this purpose, data from parental questionnaires on gestational age, birth weight and length at birth of children were compared with corresponding information collected externally during the pediatric preventive medical check-up at birth.

## Methods

### Study Population

The European IDEFICS study is a prospective cohort study aimed at identifying causes of overweight, obesity and related diseases in children (Ahrens et al., 2011). During the baseline examination (first wave: W_1_) from 2007 to 2008 and the first follow-up (second wave: W_2_) two years later, 18,773 children aged 2 to 11 years were recruited in the 8 participating European countries (Belgium, Cyprus, Estonia, Germany, Hungary, Italy, Spain, Sweden). Of these 18,773 children, 2543 were newly recruited in W_2_. Ethical approval was obtained from local ethic committees of each study center and parents provided written informed consent before their children were enrolled in the study. Examinations and sample collections were only conducted if both, parents and children, gave their consent. Upon recruitment, parental questionnaires were used to obtain information on the children's health status, living conditions, and details about pregnancy and birth. If available, the medical check-up booklets, containing information about the pregnancy outcome as recorded by the maternity care provider at birth, were also collected. A detailed description of the IDEFICS cohort can be found elsewhere (Ahrens et al., 2011, 2017).

**Fig. 1:**
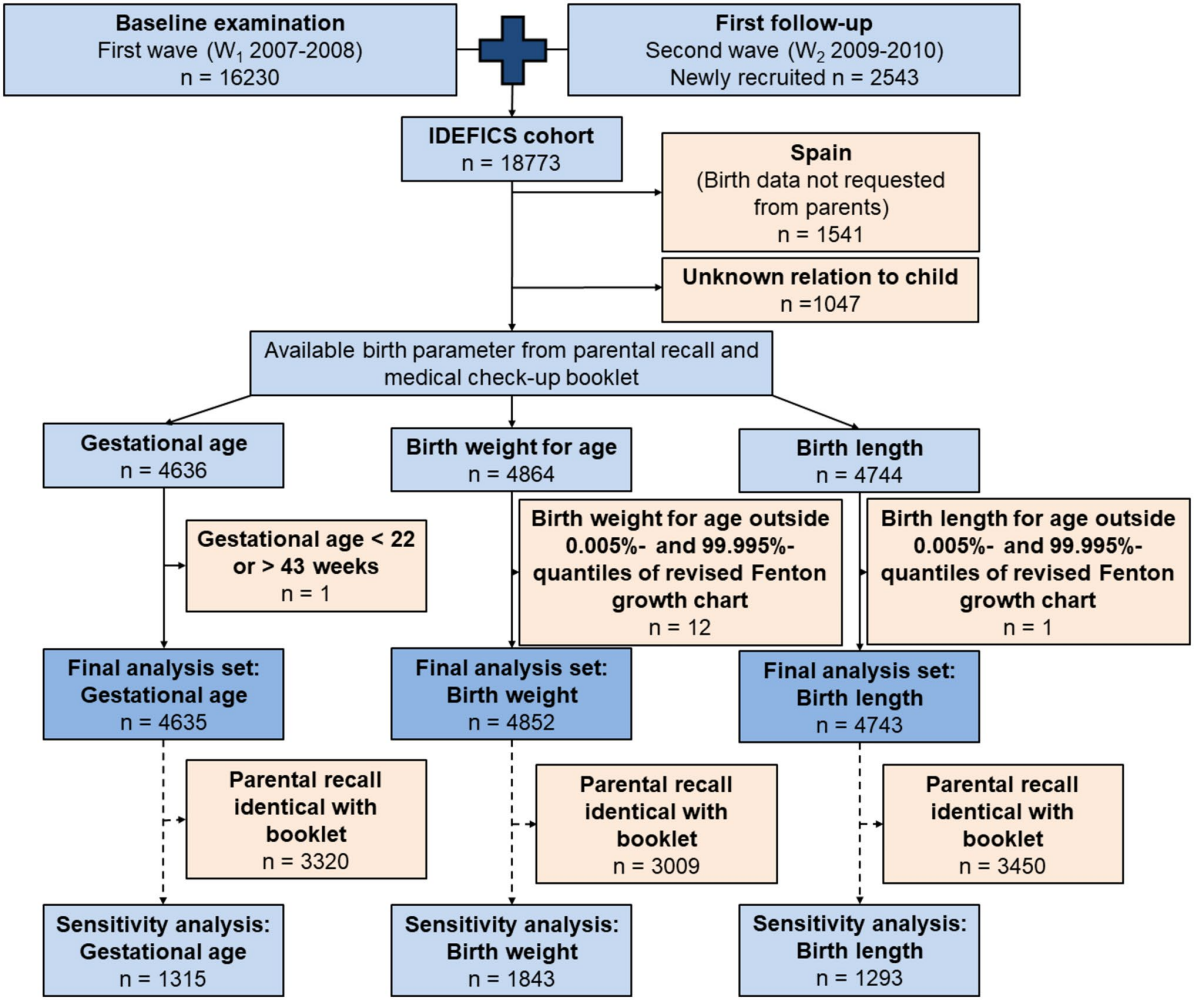
Flow chart of the study population and sample size

For the current analyses, data from Spain had to be excluded, as the birth data were not requested from the parents but taken directly from the booklets. Furthermore, children with missing information on which parent completed the questionnaire were excluded from the analyses. After removal of implausible values (gestational age < 22 or > 43 weeks, birth weight/ birth length outside the 0.005%–99.995%-quantiles (≈−/ + 4 SD) of the revised Fenton growth chart (Fenton & Kim, 2013)), children with available gestational age, birth weight or birth length from the parental questionnaire and the medical check-up booklet were included in the respective analysis set (see Fig. 1). No data from the medical check-up booklet were available for Sweden.

### Questionnaires

The questionnaires were completed by parents or legal guardians at home in handwritten form, and contained information about the living situation of the child and her/his family. Data were collected on the number of older and younger siblings the child lived with (including half-siblings or stepsiblings), on sociodemographic characteristics of the family, and on pregnancy and early childhood, including birth weight (in g) and birth length (in cm). Furthermore, parents were asked whether their child was born preterm and if so, how many weeks the child was born prematurely. Because the medical check-up booklet included the gestational age, we considered the following definitions of preterm birth: the common definition of less than 40 weeks, the World Health Organization’s (WHO) definition of less than 37 weeks (March of Dimes et al., 2012) and the definition of less than 39 weeks suggested by the work group “Defining Term Pregnancy” (Spong, 2013). Since the best agreement between the information from the questionnaire and from the booklet was obtained for the latter definition (Table 6 in Appendix A), pregnancies shorter than 39 weeks were declared preterm.

### Statistical Analysis

All statistical analyses were performed with SAS Software Version 9.4 (Copyright©2012–2016, SAS Institute Inc., Cary, NC, USA.).

First, the agreement between parental questionnaire and medical check-up booklet was examined visually by means of Bland–Altman plots (Altman & Bland, 1983). For a robust assessment of the agreement and correlation between recalled and externally recorded data and in order to maximize the comparability with other studies, various correlation coefficients (Pearson, Spearman, Kendall) as well as the intraclass correlation coefficient (ICC) for agreement with single measurements were calculated using a two-factor mixed model (McGraw & Wong, 1996). For gestational age, the correlation coefficients were additionally derived for preterm births only. Furthermore, the continuous birth parameters were manually split into categories using clinically relevant cut-offs (Adegboye & Heitmann, 2008; Spong, 2013). Cohen’s kappa was then used to assess the agreement for these categories. Since the length at birth is not commonly used in clinical practice, it was not possible to obtain clinically relevant cut-offs. Therefore, the 5% and 95% percentiles according to the WHO growth standard for children were used instead (WHO Multicentre Growth Reference Study Group, 2006).

Second, logistic regression was used to identify factors affecting the accuracy of parental recall. In line with previous studies (Adegboye & Heitmann, 2008; Tate et al., 2005), a discrepancy of more than 2 weeks in gestational age, more than 100 g in birth weight or more than 2 cm in birth length was considered inaccurate, and potential risk factors for these discrepancies were analyzed. Other clinically relevant discrepancies of more than one week for the gestational age and of more than 200 g for the birth weight were also investigated. For each of these discrepancies, we calculated a regression model with the following potentially influencing variables and adjusted for country and examination wave (W_1_ or W_2_): sex and age of the child, relationship to the child (mother, father, or a non-biological parent), SES of the parents (low, medium, high), number of siblings (including half-siblings and stepsiblings), and the examined birth parameter recorded in the medical check-up booklet. SES was measured using education level as a proxy, i.e., the highest ISCED-level (UNESCO Institute for Statistics, 2012) attained by the parents, summarized as low (levels 0–2), medium (levels 3–4) or high education (levels 5–8). Continuous covariates were assumed to have a linear influence, and if the linearity assumption did not hold, the corresponding variable was categorized.

Since the parents completed the questionnaires at home, we cannot exclude the possibility that they used the medical check-up booklet when completing the questionnaire. Therefore, we performed a sensitivity analysis in which we excluded all children for whom the parental questionnaire and the medical check-up booklet yielded identical values for the respective birth parameter.

## Results

### Study Population

The largest dataset was available for birth weight including 4852 children, 4743 children with recalled and recorded birth length were eligible, and gestational age was available for 4635 children. Compared with the complete IDEFICS cohort the three analysis datasets had a higher proportion of children from Estonia and Germany but a lower proportion of children from the other countries (see Table 1). Furthermore, compared with the complete IDEFICS cohort, more questionnaires were available from mothers, families of high SES and for single children whereas missing information on SES and the number of siblings was more common in the complete cohort.

**Table 1:** Children’s characteristics in the full IDEFICS cohort and the three analysis sets

Characteristics	Observations of children (Analysis set)			
	IDEFICS cohort	Gestational age	Birth weight	Birth length
Total	18,773 (100.0)	4635 (100.0)	4852 (100.0)	4743 (100.0)
**Examination wave (n, %)**				
W_1_	16,230 (86.5)	4022 (86.8)	4224 (87.1)	4122 (86.9)
W_2_	2543 (13.5)	613(13.2)	628 (12.9)	621 (13.1)
**Country (n, %)**				
Belgium	2396 (12.8)	481 (10.4)	522 (10.8)	516 (10.9)
Cyprus	2959 (15.8)	111 (2.4)	115 (2.4)	106 (2.2)
Estonia	2150 (11.5)	1918 (41.4)	1893 (39.0)	1881 (39.7)
Germany	2211 (11.8)	1423 (30.7)	1495 (30.8)	1489 (31.4)
Hungary	3245 (17.3)	324 (7.0)	338 (7.0)	322 (6.8)
Italy	2440 (13.0)	378 (8.2)	489 (10.1)	429 (9.0)
Spain	1541 (8.2)	0 (0.0)	0 (0.0)	0 (0.0)
Sweden	1831 (9.8)	0 (0.0)	0 (0.0)	0 (0.0)
**Child’s age (mean, SD)**				
Child’s age [in years]	6.2 (1.9)	6.1 (2.0)	6.1 (2.0)	6.1 (2.0)
**Child’s sex (n, %)**				
Male	9476 (50.5)	2310 (49.8)	2421 (49.9)	2370 (50.0)
Female	9297 (49.5)	2325 (50.2)	2431 (50.1)	2373 (50.0)
**Relation to child (n, %)**				
Missing or unclear	1112 (5.9)	0 (0.0)	0 (0.0)	0 (0.0)
Biological mother	15,347 (81.8)	4242 (91.5)	4428 (91.3)	4334 (91.4)
Biological father	2006 (10.7)	348 (7.5)	375 (7.7)	361 (7.6)
Other relationship	308 (1.6)	45 (1.0)	49 (1.0)	48 (1.0)
**Parental SES**^a^ **(n, %)**				
Missing	887 (4.7)	38 (0.8)	40 (0.8)	39 (0.8)
Low SES	1234 (6.6)	239 (5.2)	287 (5.9)	268 (5.7)
Medium SES	8175 (43.5)	2076 (44.8)	2188 (45.1)	2135 (45.0)
High SES	8477 (45.2)	2282 (49.2)	2337 (48.2)	2301 (48.5)
**Number of siblings (n, %)**				
Missing	1529 (8.1)	92 (2.0)	93 (1.9)	93 (2.0)
No siblings	3523 (18.8)	1096 (23.6)	1134 (23.4)	1112 (23.4)
1 Sibling	9246 (49.3)	2324 (50.1)	2448 (50.5)	2387 (50.3)
2 Siblings	3374 (18.0)	855 (18.4)	906 (18.7)	880 (18.6)
3 Siblings	779 (4.1)	188 (4.1)	186 (3.8)	187 (3.9)
4 + Siblings	322 (1.7)	80 (1.7)	85 (1.8)	84 (1.8)

^a^Socioeconomic status measured by the educational level as a proxy (based on ISCED)

As recorded in the medical check-up booklet, 27% of the children in the gestational age analysis set were born preterm, on average 0.7 weeks premature (standard deviation (SD) = 1.6 weeks). The average birth weight recorded in the booklet was 3413 g (SD = 565 g) and the average length at birth was 50.9 cm (SD = 2.9 cm) for the corresponding analysis datasets.

### Analysis of Agreement

**Fig. 2:**
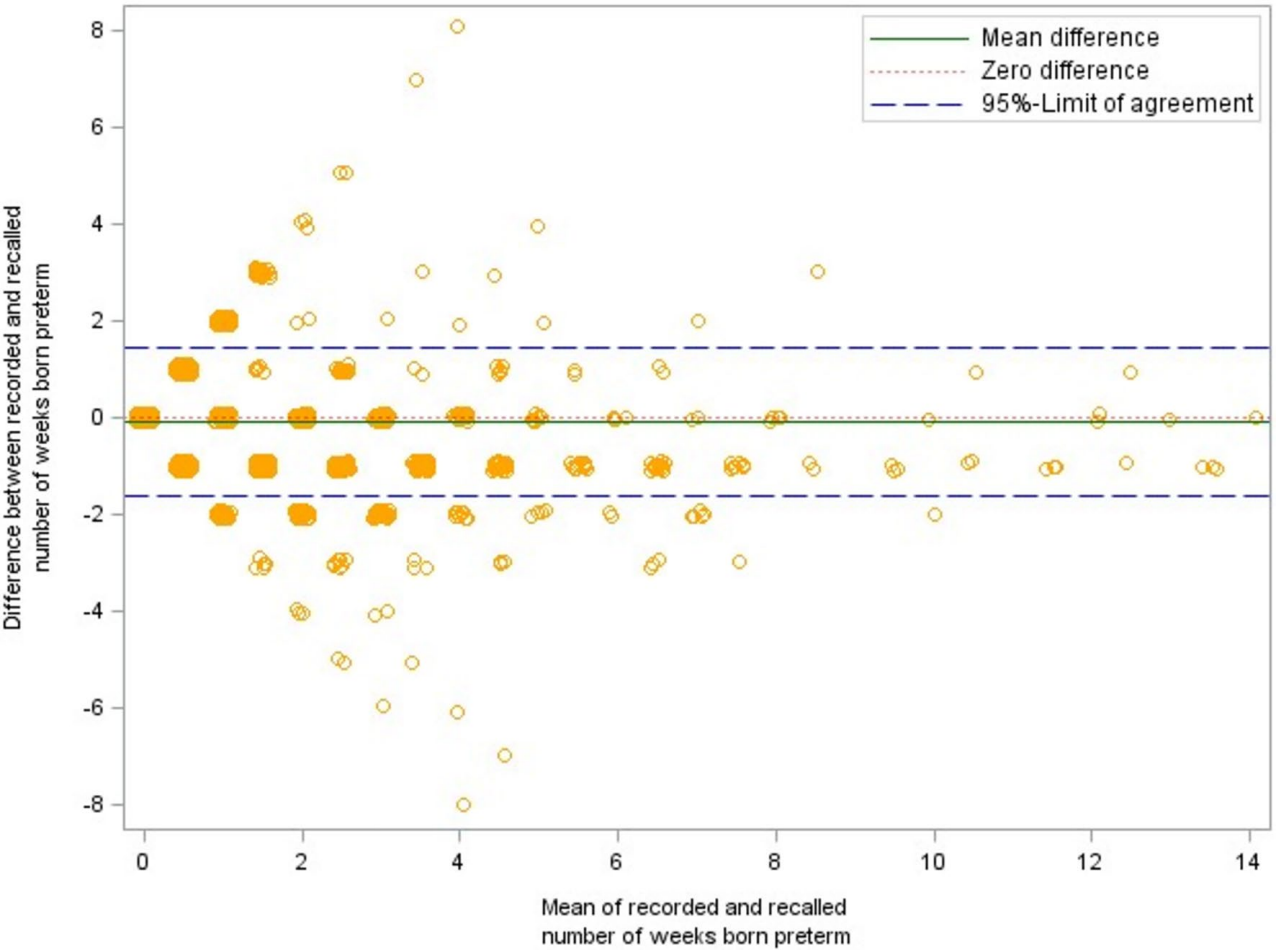
Bland–Altman plot of recorded vs. recalled gestational age with 95%-limits of agreement

**Fig. 3:**
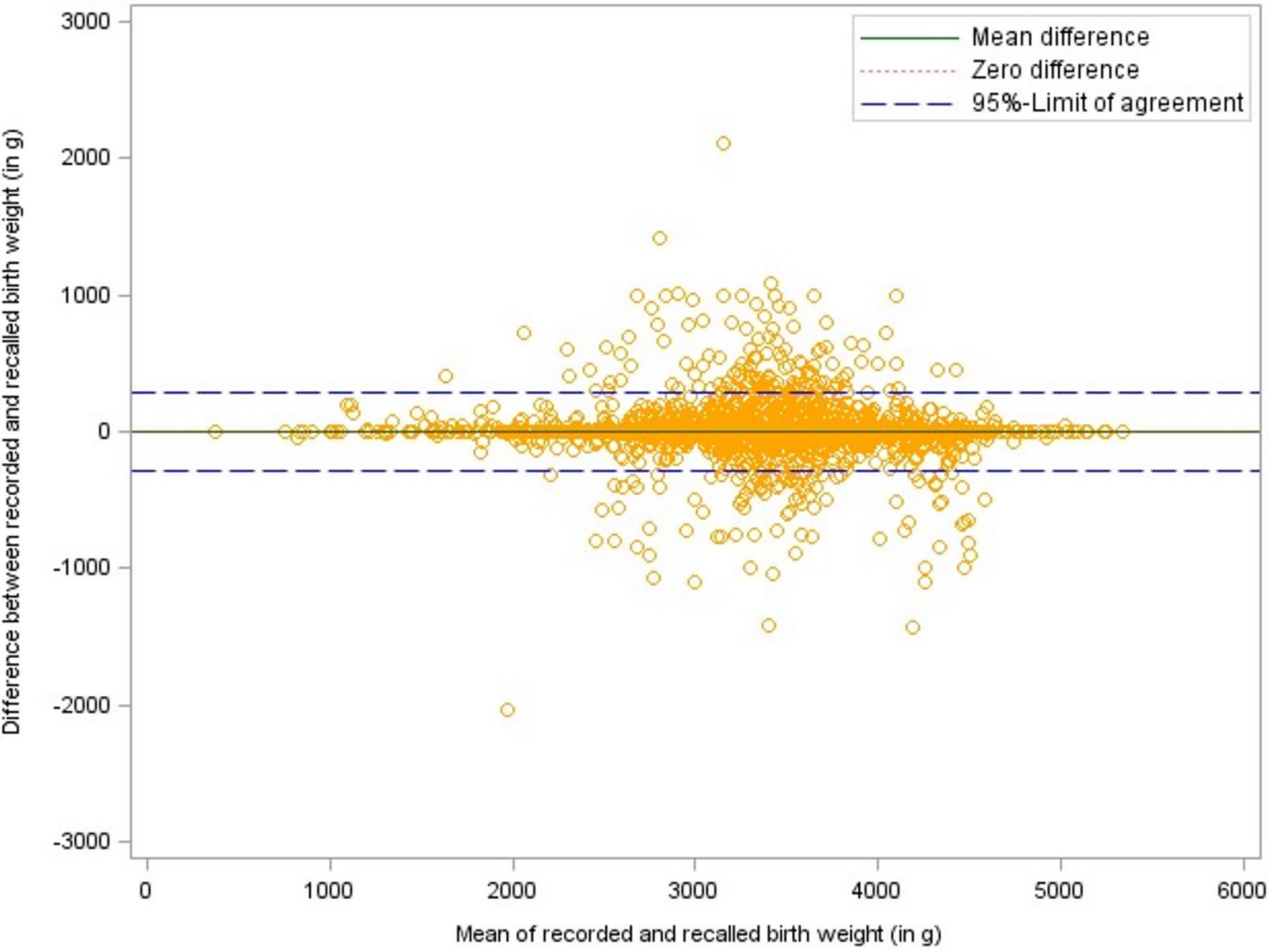
Bland–Altman plot of recorded vs. recalled birth weight with 95%-limits of agreement

**Fig. 4:**
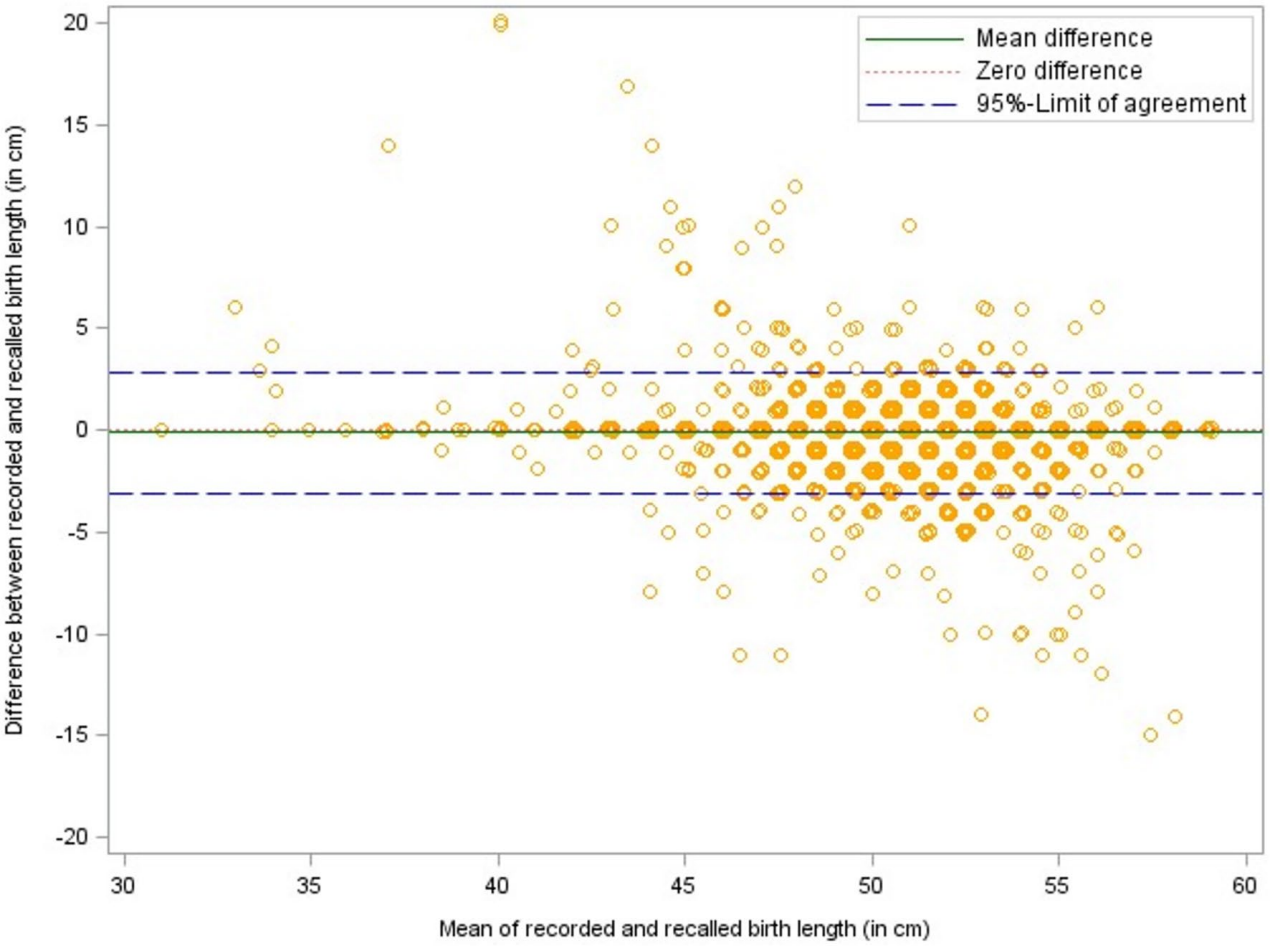
Bland–Altman plot of recorded vs. recalled birth length with 95%-limits of agreement

Parental information differed in both directions from the recorded values as become obvious from the respective Bland–Altman plots (see Fig. 2, 3, 4). On average, parental recall of the number of weeks their child was born prematurely was 0.1 weeks higher than the values in the medical check-up booklet. The birth weight reported by the parents was, on average, about 5 g lower and the reported birth length exceeded the birth length documented in the booklets by 0.1 cm. The 95% limits of agreement marked in the Bland–Altman plots were − 1.6 to 1.4 weeks for gestational age (premature births only: − 2.7 to 2.6 weeks), − 281.4 to 290.5 g for birth weight, and − 3.1 to 2.8 cm for birth length. Approximately 88% of the parents could recall their child’s birth weight within 100 g accuracy, 94% recalled birth length with 2 cm accuracy, and 99% of the parents could state within a 2 weeks accuracy how many weeks their child was born preterm (see Table 2).

**Table 2:** Frequency and percentage of discrepancies between recalled and recorded birth parameters

Gestational age (*N* = 4635)			Birth weight (*N* = 4852)			Birth length (*N* = 4743)		
Discrepancy	*n*	%	Discrepancy	*n*	%	Discrepancy	*n*	%
No difference	3320	71.6	No difference	3009	62.0	No difference	3450	72.7
1 week	1036	22.4	> 0–50 g	881	18.2	> 0–1 cm	707	14.9
2 weeks	211	4.6	> 50–100 g	381	7.9	> 1–2 cm	313	6.6
3 weeks	48	1.0	> 100–500 g	483	10.0	> 2–5 cm	212	4.5
4 weeks	9	0.2	> 500–1000 g	87	1.8	> 5–10 cm	45	0.9
5 + weeks	11	0.2	> 1000 g	11	0.2	> 10 cm	16	0.3

Table 3 shows the correlation coefficients for the data from the parental questionnaires compared with the medical check-up booklets. Pearson’s correlation coefficient for gestational age was 0.86 (95% CI 0.84–0.89) and 0.82 (95% CI 0.79–0.86) when only preterm births were considered. For birth weight, Pearson’s correlation coefficient was 0.97 (95% CI 0.96–0.97) and for birth length, 0.85 (95% CI 0.83–0.88) (see Table 3). The ICCs provided only slightly lower values than the Pearson’s correlation coefficients: 0.85 (95% CI 0.84–0.86) for gestational age (preterm births only: 0.80 (95% CI 0.78–0.82)), 0.97 (95% CI 0.96–0.97) for birth weight and 0.85 (95% CI 0.84–0.86) for birth length. For the sensitivity analyses described above, i.e., when observations with exactly matching data were excluded, the correlation coefficients were lower compared to the values for the full analysis sets (Table 7 in Appendix B).

**Table 3:** Correlation between recalled and recorded birth parameters

Birth parameter		Correlation coefficient	Value	95% CI
Gestational age [weeks born preterm]	All data (*N* = 4635)	Pearson Correlation	0.86	(0.84, 0.89)
		Spearman's Rho	0.68	(0.65, 0.70)
		Kendall's Tau-b	0.65	(0.63, 0.67)
		ICC^a^	0.85	(0.84, 0.86)
	Preterm only (*N* = 1255)	Pearson Correlation	0.82	(0.79, 0.86)
		Spearman's Rho	0.66	(0.62, 0.70)
		Kendall's Tau-b	0.59	(0.56, 0.63)
		ICC^a^	0.80	(0.78, 0.82)
Birth weight [in g]	All data (*N* = 4852)	Pearson Correlation	0.97	(0.96, 0.97)
		Spearman's Rho	0.96	(0.96, 0.97)
		Kendall's Tau-b	0.91	(0.90, 0.91)
		ICC^a^	0.97	(0.96, 0.97)
Birth length [in cm]	All data (*N* = 4743)	Pearson Correlation	0.85	(0.83, 0.88)
		Spearman's Rho	0.88	(0.87, 0.89)
		Kendall's Tau-b	0.82	(0.80, 0.83)
		ICC^a^	0.85	(0.84, 0.86)

^a^*ICC* Intraclass correlation coefficient—single measurements, two-factor mixed model, agreement. The ICC was calculated according to McGraw and Wong (1996) based on the restricted maximum likelihood estimates from the SAS procedure PROC MIXED

Regarding agreement between parental questionnaires and medical check-up booklets for clinically relevant categories, the proportion of matching classifications was highest for birth weight with 98.2% (see Table 4). For gestational age, this proportion was 80.4% and for birth length 93.0%. The agreement for gestational age could be classified as moderate (Cohen’s kappa: 0.52) and as good for birth weight and birth length (Cohen’s kappa: birth weight 0.94, birth length 0.80). Table 4 shows that for almost all categories (according to the child’s medical check-up booklet), most children were also assigned to this category based on the parentally recalled data. The only exception are early births (gestational age 37–39 weeks) which are more often assigned to term and postterm births based on the parental information. Regarding the classification in preterm and term births, 816 births (65.0% of all preterm births) were correctly classified as preterm, resulting in an overall agreement of 85.0% and a Cohen’s kappa of 0.60 (95% CI 0.58–0.63).

**Table 4:** Agreement between recalled and recorded birth parameters for categories based on clinically relevant cut-offs

**Gestational age [agreement 80.4%]**				
	**Recorded (n, %)**			**Cohen’s kappa** **(value, 95% CI)**
**Recalled**	*Delivered in time or later* *[> = 39 weeks]*	*Early birth* *[37– < 39 weeks]*	*Late to extremely preterm* *[< 37 weeks]*	0.52 (0.49, 0.54)
*Delivered in time or later* *[> = 39 weeks]*	3123 (67.4%)	410 (8.8%)	29 (0.6%)	
*Early birth* *[37– < 39 weeks]*	245 (5.3%)	347 (7.5%)	18 (0.4%)	
*Late to extremely preterm* *[< 37 weeks]*	12 (0.3%)	193 (4.2%)	258 (5.6%)	
**Birth weight [agreement 98.2%]**				
	**Recorded (n, %)**			**Cohen’s Kappa** **(value, 95% CI)**
**Recalled**	*Low birth weight* *[< 2500 g]*	*Normal birth weight* *[2500-4000 g]*	*High birth weight* *[> 4000 g]*	0.94 (0.93, 0.95)
*Low birth weight* *[< 2500 g]*	218 (4.5%)	23 (0.5%)	1 (0.0%)	
*Normal birth weight* *[2500–4000 g]*	21 (0.4%)	3922 (80.8%)	28 (0.6%)	
*High birth weight* *[> 4000 g]*	0 (0.0%)	15 (0.3%)	624 (12.9%)	
**Birth length [agreement 93.0%]**				
	**Recorded (n, %)**			**Cohen’s Kappa** **(value, 95% CI)**
**Recalled**	*Low birth length* *[< 48 cm]*	*Normal birth length* *[48-53 cm]*	*High birth length* *[> 53 cm]*	0.80 (0.78, 0.82)
*Low birth length* *[< 48 cm]*	304 (6.4%)	71 (1.5%)	2 (0.0%)	
*Normal birth length* *[48-53 cm]*	65 (1.4%)	3591 (75.7%)	64 (1.3%)	
*High birth length* *[> 53 cm]*	4 (0.1%)	125 (2.6%)	517 (10.9%)	

### Identification of Potential Risk Factors for Discrepancies Between Recalled and Recorded Birth Parameters

In the logistic regression models for gestational age, a trend for increased risk of discrepancies of more than 2 weeks was observed with increasing number of siblings (see Table 5). In addition, parents with medium or low SES had an approximately two-fold chance of having discrepancies of more than 2 weeks compared to parents with high SES. Furthermore, the risk of discrepancies of more than 2 weeks increased with the number of weeks the child was born preterm. Similar results were observed for the sensitivity analysis (see Table 5). Modelling discrepancies of more than one week, the risk of discrepancies increased only with the number of weeks the child was born preterm (Table 8 in Appendix B).

**Table 5:** Odds ratios (OR) and 95% confidence intervals (95% CI) for discrepancies between recalled and recorded birth parameters

	Main analysis			Sensitivity analysis		
Covariates	*N*	*OR*^a^	95%* CI*^a^	*N*	*OR*^a^	95%* CI*^a^
*Outcome: Discrepancies of > 2 weeks for the gestational age*						
**Child’s age**						
Increase of one year	4635	1.0	(0.9–1.2)	1315	1.1	(0.9–1.2)
**Child’s sex**						
Female	2325	1.0	Reference	637	1.0	Reference
Male	2310	1.0	(0.6–1.6)	678	0.9	(0.5–1.5)
**Relation to child**						
Biological mother	4242	1.0	Reference	1175	1.0	Reference
Biological father	348	1.3	(0.6–2.9)	128	1.2	(0.5–2.5)
Other relationship	45	NE^c^	NE^c^	12	NE^c^	NE^c^
**Parental SES**^b^						
High SES	2282	1.0	Reference	636	1.0	Reference
Medium SES	2076	2.0	(1.1–3.6)	593	2.0	(1.1–3.6)
Low SES	239	2.1	(0.7–5.9)	72	2.0	(0.7–5.9)
**Number of siblings**						
No siblings	1096	1.0	reference	281	1.0	reference
1 Sibling	2324	1.3	(0.6–2.6)	663	1.2	(0.6–2.6)
2 Siblings	855	1.2	(0.5–2.9)	246	1.3	(0.5–3.0)
3 Siblings	188	3.3	(1.1–9.7)	73	2.3	(0.8–6.8)
4 + Siblings	80	5.4	(1.4–20.2)	25	6.3	(1.7–23.6)
**Weeks born preterm**						
Increase of one week	4635	1.5	(1.3–1.6)	1315	1.3	(1.1–1.4)
*Outcome: Discrepancies of > 100 g for the birth weight*						
**Child’s age**						
Increase of one year	5104	1.1	(1.0–1.2)	1843	1.1	(1.0–1.2)
**Child’s sex**						
Female	2431	1.0	Reference	905	1.0	Reference
Male	2421	1.2	(1.0–1.5)	938	1.2	(1.0–1.4)
**Relation to child**						
Biological mother	4428	1.0	Reference	1648	1.0	Reference
Biological father	375	1.1	(0.8–1.5)	170	0.8	(0.6–1.2)
Other relationship	49	3.4	(1.7–6.7)	25	3.0	(1.2–7.4)
**Parental SES**^b^						
High SES	2337	1.0	Reference	983	1.0	Reference
Medium SES	2188	1.3	(1.1–1.6)	755	1.4	(1.2–1.8)
Low SES	287	2.3	(1.6–3.4)	92	2.4	(1.5–4.0)
**Number of siblings**						
No siblings	1134	1.0	Reference	400	1.0	Reference
1 Sibling	2448	1.2	(1.0–1.6)	927	1.1	(0.8–1.4)
2 Siblings	906	1.5	(1.1–1.9)	365	1.2	(0.8–1.6)
3 Siblings	186	2.0	(1.2–3.1)	76	1.4	(0.8–2.3)
4 + Siblings	85	4.4	(2.5–7.7)	39	3.1	(1.5–6.6)
**Birth weight**						
Normal birth weight (2500-4000 g)	3960	1.0	Reference	1485	1.0	Reference
Low birth weight (< 2500 g)	239	1.3	(0.9–1.9)	92	1.2	(0.8–1.9)
High birthweight (> 4000 g)	653	0.9	(0.7–1.2)	266	1.1	(0.8–1.4)
*Outcome: Discrepancies of > 2 cm for the birth length*						
**Child’s age**						
Increase of one year	4743	1.0	(0.9–1.1)	1293	0.9	(0.9–1.0)
**Child’s sex**						
Female	2373	1.0	Reference	637	1.0	Reference
Male	2370	0.9	(0.7–1.2)	656	0.9	(0.7–1.2)
**Relation to child**						
Biological mother	4334	1.0	Reference	1167	1.0	Reference
Biological father	361	1.5	(1.0–2.3)	108	1.6	(1.0–2.5)
Other relationship	48	1.1	(0.3–4.7)	18	0.6	(0.1–2.9)
**Parental SES**^b^						
High SES	2301	1.0	Reference	661	1.0	Reference
Medium SES	2135	1.0	(0.8–1.4)	559	1.1	(0.8–1.5)
Low SES	268	0.9	(0.5–1.6)	62	1.0	(0.5–1.9)
**Number of siblings**						
No siblings	1112	1.0	Reference	269	1.0	Reference
1 Sibling	2387	1.0	(0.7–1.4)	662	0.9	(0.6–1.3)
2 Siblings	880	1.3	(0.9–2.0)	265	1.0	(0.6–1.5)
3 Siblings	187	1.1	(0.5–2.3)	42	0.8	(0.3–1.9)
4 + Siblings	84	2.8	(1.2–6.4)	27	2.0	(0.8–5.0)
**Birth length**						
Normal birth length (48–53 cm)	3787	1.0	Reference	1048	1.0	Reference
Low birth length (< 48 cm)	373	3.0	(2.1–4.2)	117	3.4	(2.2–5.3)
High birth length (> 53 cm)	583	2.0	(1.4–3.0)	128	2.2	(1.4–3.4)

^a^Adjusted for the examination wave, examination country and the factors shown in the respective subpart of the table

^b^Socioeconomic status measured by the educational level as a proxy (based on ISCED)

^c^Not estimable with maximum likelihood method due to separation of data points. Estimates obtained with Firth’s penalized likelihood can be found in Table 9 in Appendix B

Regarding the outcome birth weight, the models for discrepancies of more than 100 g and 200 g between recalled and recorded value showed similar associations. Non-biological parents had a higher chance of discrepancies than mothers (see Table 5 and Table 8 in Appendix B). Furthermore, parents with low SES were twice as likely to report deviating birth weights as parents with high SES (see Table 5 and Table 8 in Appendix B). In addition, the likelihood of discrepancies greater than 100 g and 200 g was increased for children with more siblings and increased with increasing age of the child (see Table 5 and Table 8 in Appendix B). Again, similar results were observed in the sensitivity analyses.

Regarding birth length, a strong association between measured birth length and the accuracy of parental recall could be observed, i.e., the chance of discrepancies was lower for children with average birth length (see Table 5). In addition, fathers were 1.5 times more likely to report a birth length that deviated by more than 2 cm from the one recorded in the booklet compared to mothers. Furthermore, there was a slightly increased chance of discrepancies for children living with more than three siblings. However, this tendency was less pronounced in the sensitivity analyses (see Table 5).

## Discussion

Our analyses have shown that the accuracy of parental recall of gestational age, birth weight and birth length is generally good: compared with the birth weight documented in the medical booklet 88% of the parents accurately reported the birth weight of their child of up to 100 g, which is in line with the results of previous studies in the UK. O'Sullivan et al. (2000) were able to show that 85% of the mothers of 6 to 15-year-old children could accurately recall the weight up to 100 g and in another study, but in younger children at 9 months of age, a 92% accuracy within 100 g was observed (Tate et al., 2005). The slight variations in accuracy might be explained by the age difference since our results indicated that the reliability of the parentally recalled birth weight decreases with increasing age of the children. Furthermore, our calculated 95% limits of agreement for birth weight of − 281.4 to 290.5 g also match the results of previous studies where 95% limits of agreement of − 285 and 284.5 g have been reported (Adegboye & Heitmann, 2008). Almost all parents (99%) recalled gestational age with a discrepancy of two weeks or less and the 95% limits of agreement were − 1.6 to 1.4 weeks. In a similar study from Denmark a lower proportion of 86% and 95% limits of agreement from − 2.1 to 2.6 weeks could be observed (Adegboye & Heitmann, 2008). One explanation for the higher accuracy in the IDEFICS study could be that the questionnaires did not consider whether and how many weeks the child was born postterm. Thus, discrepancies could only be observed for premature births. Parentally recalled birth length showed a high agreement with the medical check-up booklet, 94% of the parentally recalled lengths were accurate within 2 cm. The logistic regression analysis revealed a lower chance for discrepancies of more than 2 cm for children with average birth length (48–53 cm). This might be attributed to the limited range of birth lengths for this category, compared to the low (< 48 cm) and high (> 53 cm) birth length category.

Several factors may influence the accuracy of parental recall. For example, discrepancies between recalled and recorded gestational age were more common in families with low or medium SES. This is in line with the results of previous studies that found an association between the accuracy of maternal recall and the mother’s educational level (Colacce et al., 2020) and marital status (Adegboye & Heitmann, 2008), which could be considered as a proxy for socioeconomic class. Our analysis revealed a similar association between the accuracy of the recall and the SES for the birth weight, which has also been reported by Tate et al. (2005). Additionally, we observed that discrepancies between recalled and reported birth weight were more likely for non-biological parents, older children and for children with many siblings. Previous studies have shown similar results for children with older siblings (Adegboye & Heitmann, 2008; Tate et al., 2005). Interestingly, in contrast to another study with parents of adult children (Kassem et al., 2018), we did not observe a pronounced difference in the accuracy of recalled birth weight by fathers compared to mothers.

### Strengths and Limitations

The IDEFICS study enabled us to analyze the reliability of birth parameters recalled by parents using a large dataset from an international population and to identify several factors that may influence recall accuracy. The size and diversity of the study population stand out as particular strengths. Nonetheless, comparison of the birth parameters was only possible for a subset of the IDEFICS population, which had a higher proportion of mothers, families of high SES and single children than the complete cohort. Since the latter two characteristics were associated with a higher accuracy of the recalled birth parameters, the accuracy of the parental recall in the complete cohort might be slightly lower than in our analysis sets. Furthermore, it is likely that the missingness of the recalled and recorded birth parameters is related to the same factors that were found to be associated with the reliability of the recall. However, this should not have biased the estimated regression coefficients since it is unlikely that the missingness depends on the value of the birth parameter (Little & Rubin, 2002).

One major limitation of our study is that gestational age was differently asked for by the parental questionnaire and the medical check-up booklet. Thus, we had to choose a definition for preterm birth that may bias the results and it was not possible to examine discrepancies for postterm births. Further, we decided to exclude extreme values, since these values would most likely also be excluded during plausibility checks in practice. However, since only few values (n = 1 or n = 12, see Fig. 1) were excluded this should not have distorted the results.

Since the questionnaires were completed at home it cannot be ruled out that the parents used the medical check-up booklets when completing the questionnaire. Thus, in a study where the parents do not have access to the recorded data the accuracy of the parentally reported birth data could be lower than in our analyses. We considered this fact by conducting sensitivity analyses, which inevitably resulted in somewhat attenuated agreements but did not substantially change the tendency of results. However, since many parents recalled identical values to the medical check-up booklet, the sample sizes were markedly lower than in the main analysis, which resulted in wider confidence intervals for the odds ratios, in particular for groups with limited sample size (see Table 5).

## Conclusion

Our results indicate that parental recall of birth parameters is generally reliable. However, accuracy depends on the interviewed parent and the child’s living situation (e.g., SES, number of siblings). This should be taken into consideration when using parentally recalled birth data in studies of child health.

## Data Availability

Due to the prospective nature of this ongoing cohort study, the full anonymization of study data is ruled out and use of data requires a mutual agreement between our study consortium and interested third parties on a case-by-case basis. For corresponding requests, please contact the study coordinator (ahrens@leibniz-bips.de).

## Appendix A: Definitions preterm birth

See Table 6.

**Table 6:** Agreement between recalled and recorded gestational age for the different definitions of preterm birth

Accuracy within …	Preterm < 37 weeks		Preterm < 39 weeks		Preterm < 40 weeks	
	*n*	%	*n*	%	*n*	%
0 weeks	3539	76.4	3320	71.6	2928	63.2
1 week	3849	83.0	4356	94.0	4101	88.5
2 weeks	4265	92.0	4567	98.5	4510	97.3
3 weeks	4555	98.3	4615	99.6	4593	99.1
4 weeks	4614	99.5	4624	99.8	4623	99.7
5 + weeks	4635	100.0	4635	100.0	4635	100.0

## Appendix B: Supplementary Analysis

See Tables 7, 8 and 9.

**Table 7:** Correlations and 95% limits of agreement (LOA) in the sensitivity analyses

Birth parameter		Statistic	Value	95% CI or 95% LOA
Gestational age [weeks born preterm]	Sensitivity analysis (*N* = 1315)	Pearson Correlation	0.74	(0.69, 0.79)
		Spearman's Rho	0.42	(0.37, 0.48)
		Kendall's Tau-b	0.36	(0.31, 0.41)
		ICC^a^	0.72	(0.66, 0.76)
		Average difference^b^ with 95% LOA	− 0.32	(− 3.15, 2.52)
Birth weight [in g]	Sensitivity analysis (*N* = 1843)	Pearson Correlation	0.92	(0.90, 0.93)
		Spearman's Rho	0.90	(0.89, 0.92)
		Kendall's Tau-b	0.78	(0.76, 0.80)
		ICC^a^	0.92	(0.91, 0.92)
		Average difference^b^ with 95% LOA	12.03	(− 451.62, 475.68)
Birth length [in cm]	Sensitivity analysis (*N* = 1293)	Pearson Correlation	0.57	(0.51, 0.64)
		Spearman's Rho	0.57	(0.52, 0.61)
		Kendall's Tau-b	0.45	(0.42, 0.49)
		ICC^a^	0.55	(0.51, 0.59)
		Average difference^b^ with 95% LOA	− 0.41	(− 6.03, 5.21)

^a^ICC: Intraclass correlation coefficient—single measurements, two-factor mixed model, agreement. The ICC was calculated according to McGraw and Wong (1996) based on the restricted maximum likelihood estimates from the SAS procedure PROC MIXED

^b^Difference calculated as recorded minus recalled birth parameter

**Table 8:** Odds ratios (OR) and 95% confidence intervals (95% CI) for further discrepancies between recalled and recorded birth parameters

	Outcome: discrepancies of > 1 week for the gestational age			Outcome: discrepancies of > 200 g for the birth weight		
Covariates	*N*	*OR* ^a^	95%* CI*^a^	*N*	*OR* ^a^	95%* CI*^*a*^
**Child’s age**						
Increase of one year	4635	1.0	(0.9–1.0)	5104	1.1	(1.1–1.2)
**Child’s sex**						
Female	2325	1.0	Reference	2431	1.0	Reference
Male	2310	1.1	(0.9–1.4)	2421	1.2	(1.0–1.5)
**Relation to child**						
Biological mother	4242	1.0	Reference	4428	1.0	Reference
Biological father	348	1.5	(1.0–2.3)	375	0.7	(0.4–1.2)
Other relationship	45	1.2	(0.3–4.9)	49	3.3	(1.5–7.5)
**Parental SES** ^b^						
High SES	2282	1.0	Reference	2337	1.0	Reference
Medium SES	2076	1.0	(0.7–1.3)	2188	1.3	(1.0–1.7)
Low SES	239	1.2	(0.7–2.2)	287	2.4	(1.5–4.0)
**Number of siblings**						
No siblings	1096	1.0	Reference	1134	1.0	Reference
1 Sibling	2324	0.9	(0.7–1.3)	2448	1.3	(0.9–1.7)
2 Siblings	855	1.0	(0.6–1.4)	906	1.4	(0.9–2.0)
3 Siblings	188	1.9	(1.1–3.3)	186	1.2	(0.6–2.4)
4 + Siblings	80	1.6	(0.6–4.1)	85	4.2	(2.1–8.1)
**Weeks born preterm**						
Increase of one week	4635	1.5	(1.4–1.6)	/^c^		
**Birth weight**						
Normal birth weight (2500–4000 g)	/^c^			3960	1.0	Reference
Low birth weight (< 2500 g)	/^c^			239	1.2	(0.7–2.0)
High birth weight (> 4000 g)	/^c^			653	0.8	(0.6–1.2)

^a^Adjusted for the examination wave, examination country and the factors shown in the respective subpart of the table

^b^Socioeconomic status measured by the educational level as a proxy (based on ISCED)

^c^Not included in the regression model for the outcome

**Table 9:** Odds ratios (OR) and 95% confidence intervals (95% CI) for discrepancies for the gestational age using Firth’s penalized likelihood

	Main analysis			Sensitivity analysis		
Covariates	*N*	*OR* ^a^	95% *CI*^b^	*N*	*OR* ^a^	95% *CI*^b^
*Outcome: Discrepancies of > 2 weeks for the gestational age*						
**Child’s age**						
Increase of one year	4635	1.1	(0.9–1.2)	1315	1.1	(0.9–1.2)
**Child’s sex**						
Female	2325	1.0	Reference	637	1.0	Reference
Male	2310	1.0	(0.6–1.6)	678	0.9	(0.5–1.5)
**Relation to child**						
Biological mother	4242	1.0	Reference	1175	1.0	Reference
Biological father	348	1.4	(0.6–2.8)	128	1.2	(0.5–2.4)
Other relationship	45	1.3	(0.0–9.7)	12	1.1	(0.0–9.5)
**Parental SES** ^c^						
High SES	2282	1.0	Reference	636	1.0	Reference
Medium SES	2076	2.0	(1.1–3.5)	593	2.0	(1.1–3.6)
Low SES	239	2.2	(0.7–5.7)	72	2.1	(0.7–5.6)
**Number of siblings**						
No siblings	1096	1.0	Reference	281	1.0	Reference
1 Sibling	2324	1.2	(0.6–2.6)	663	1.2	(0.6–2.6)
2 Siblings	855	1.2	(0.5–2.9)	246	1.3	(0.5–3.0)
3 Siblings	188	3.4	(1.1–9.3)	73	2.3	(0.8–6.5)
4 + Siblings	80	5.6	(1.4–18.7)	25	6.3	(1.6–21.4)
**Weeks born preterm**						
Increase of one week	4635	1.5	(1.3–1.6)	1315	1.3	(1.1–1.4)

^a^Firth’s penalized likelihood estimates adjusted for the examination wave, examination country and the factors shown in the respective subpart of the table

^b^Confidence interval based on the likelihood ratio

^c^Socioeconomic status measured by the educational level as a proxy (based on ISCED)
